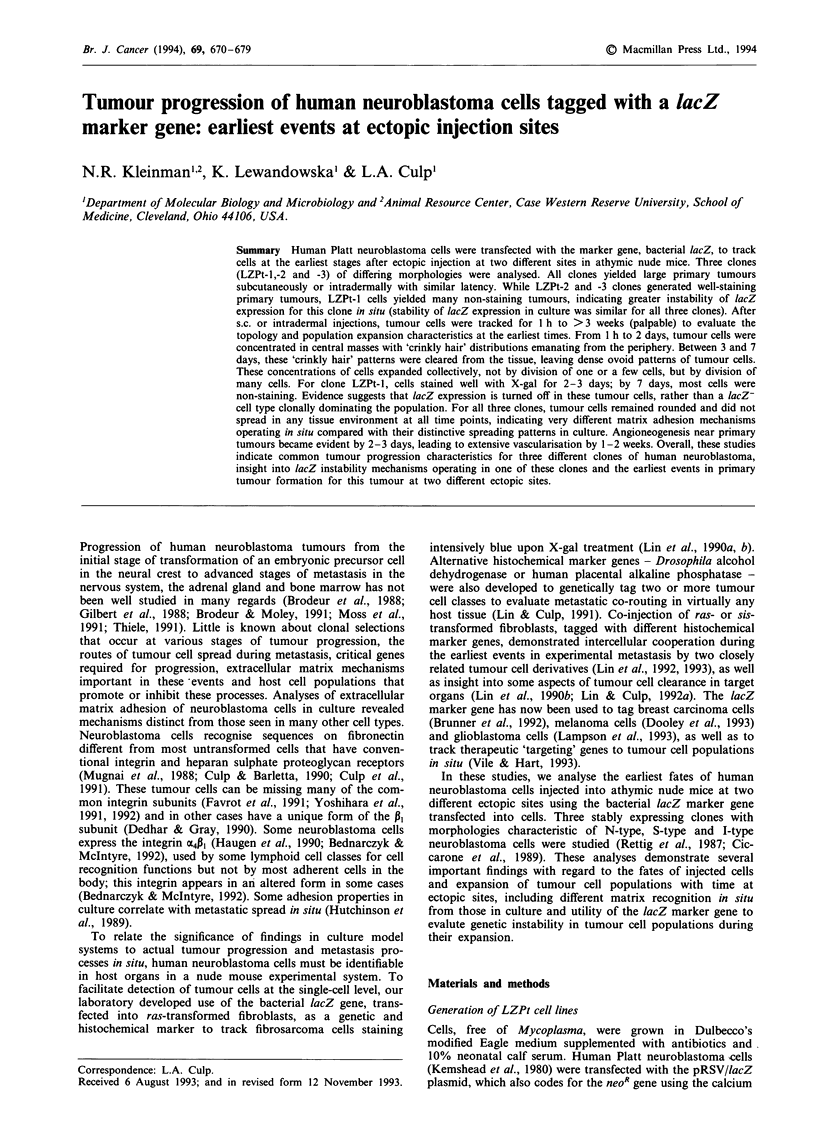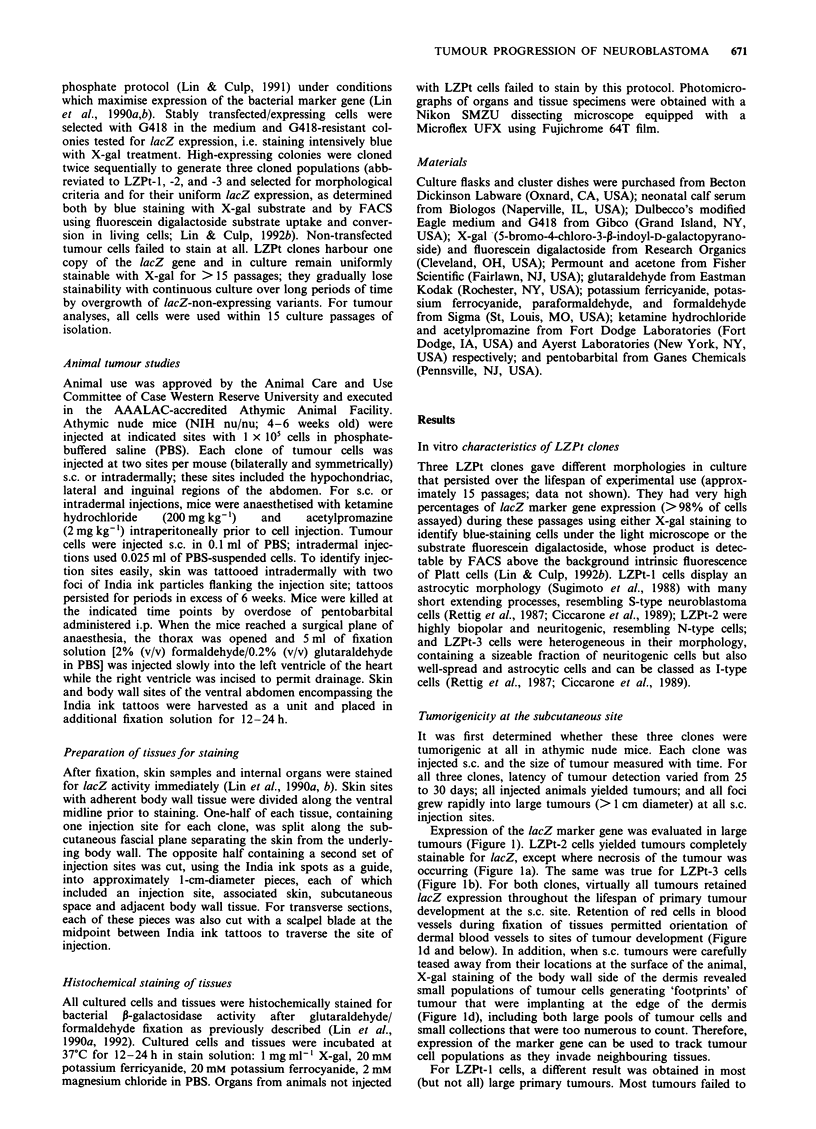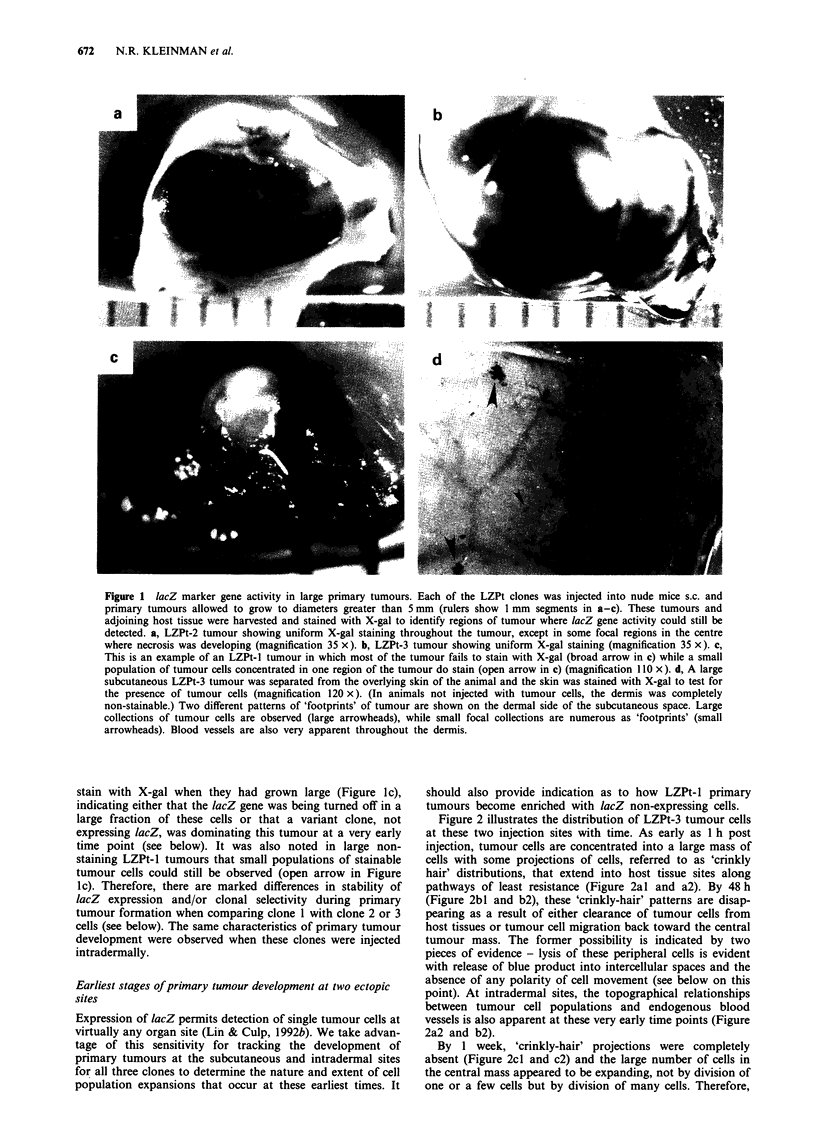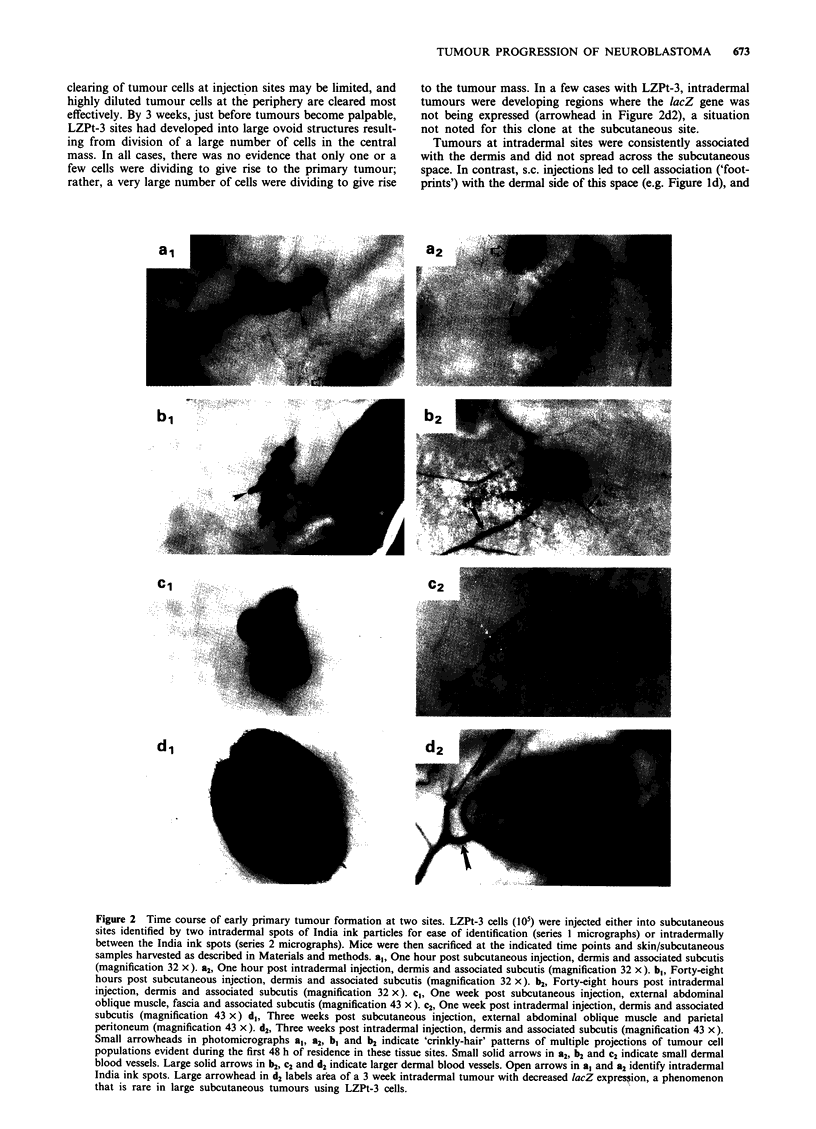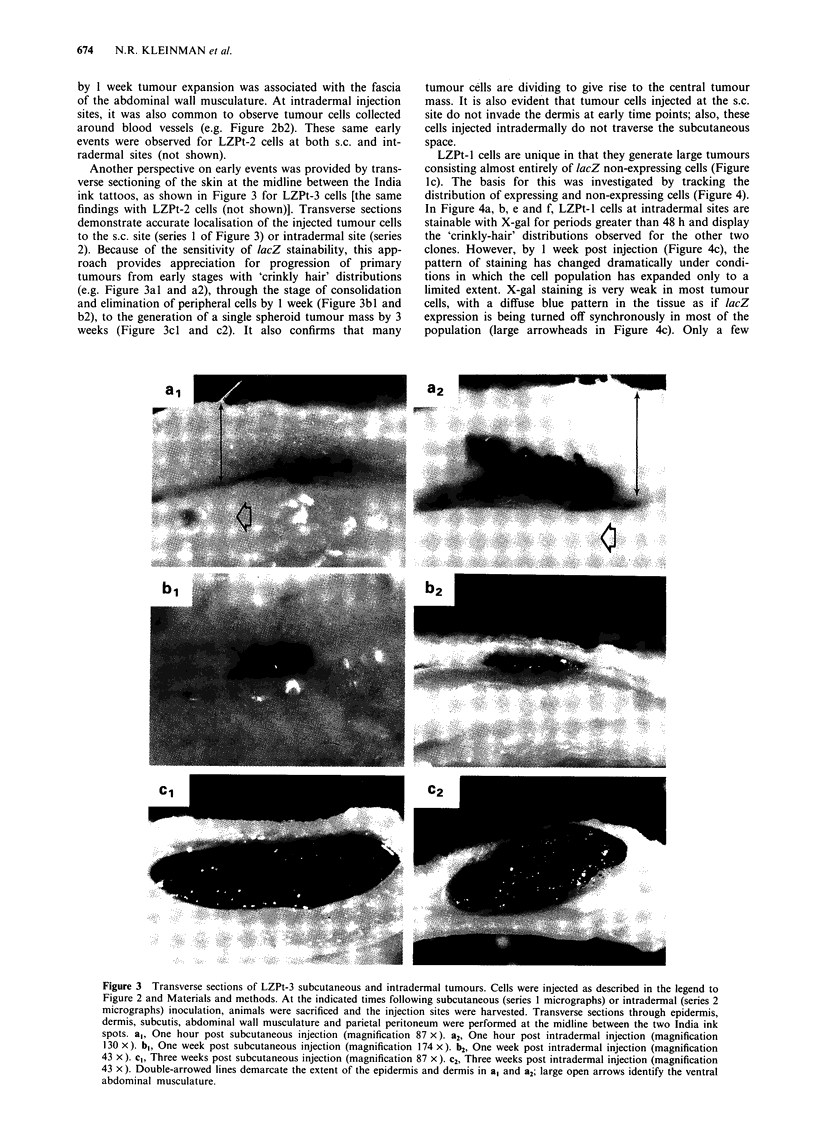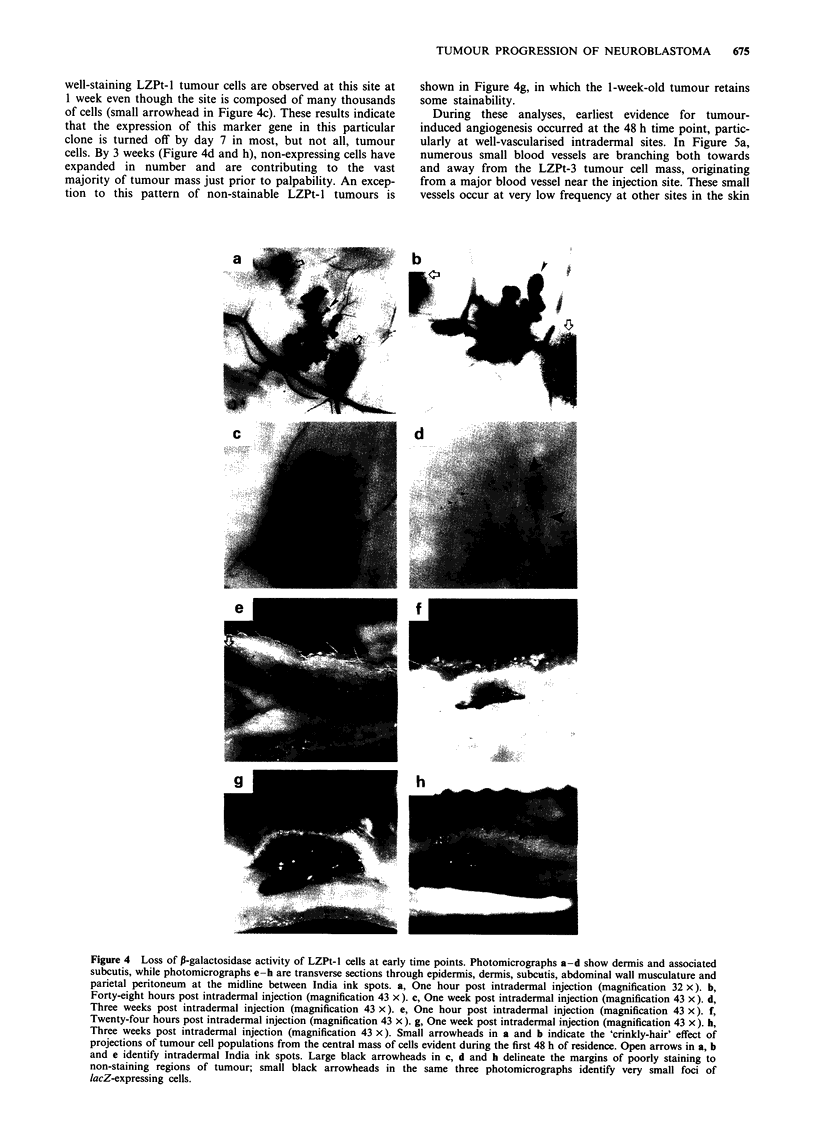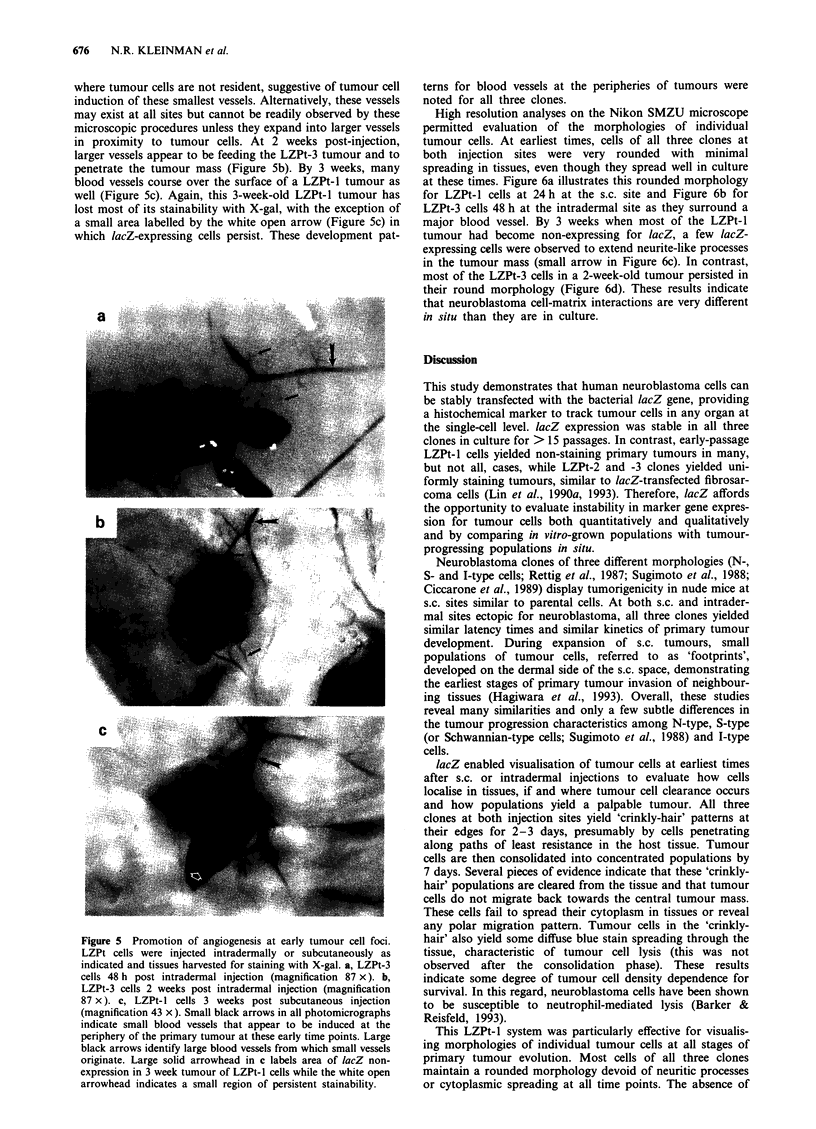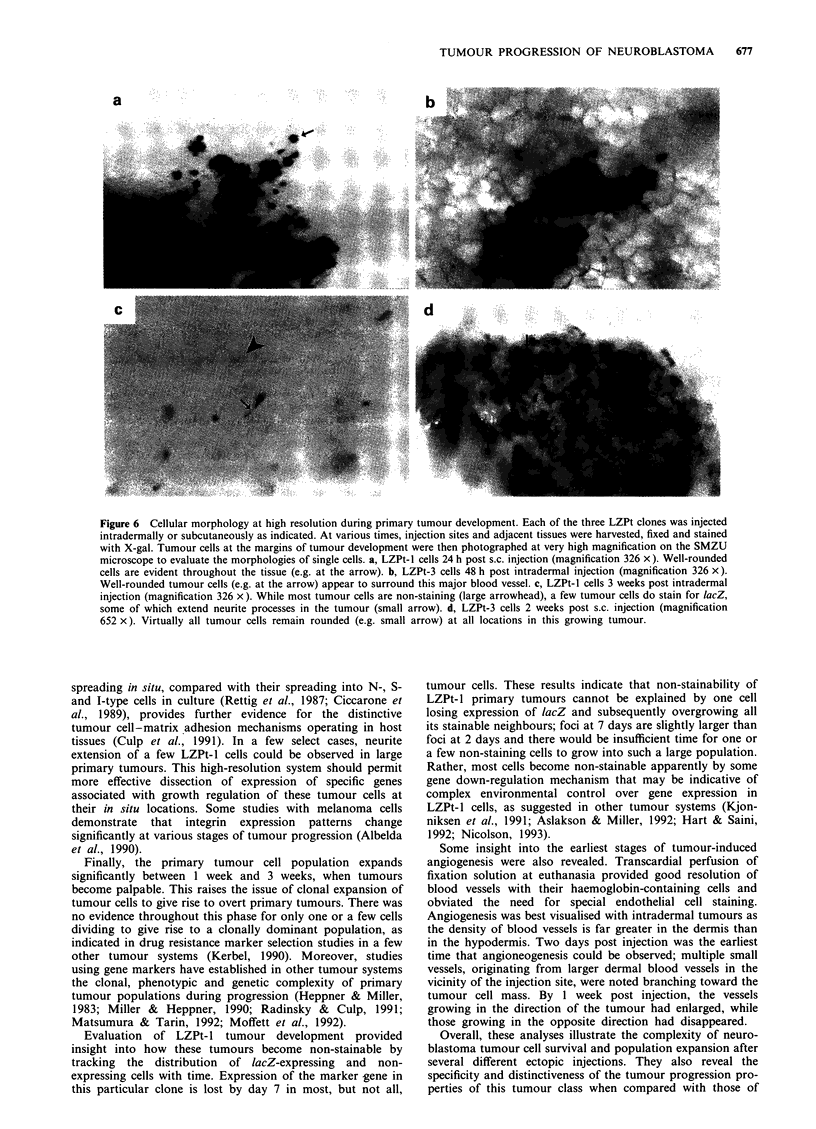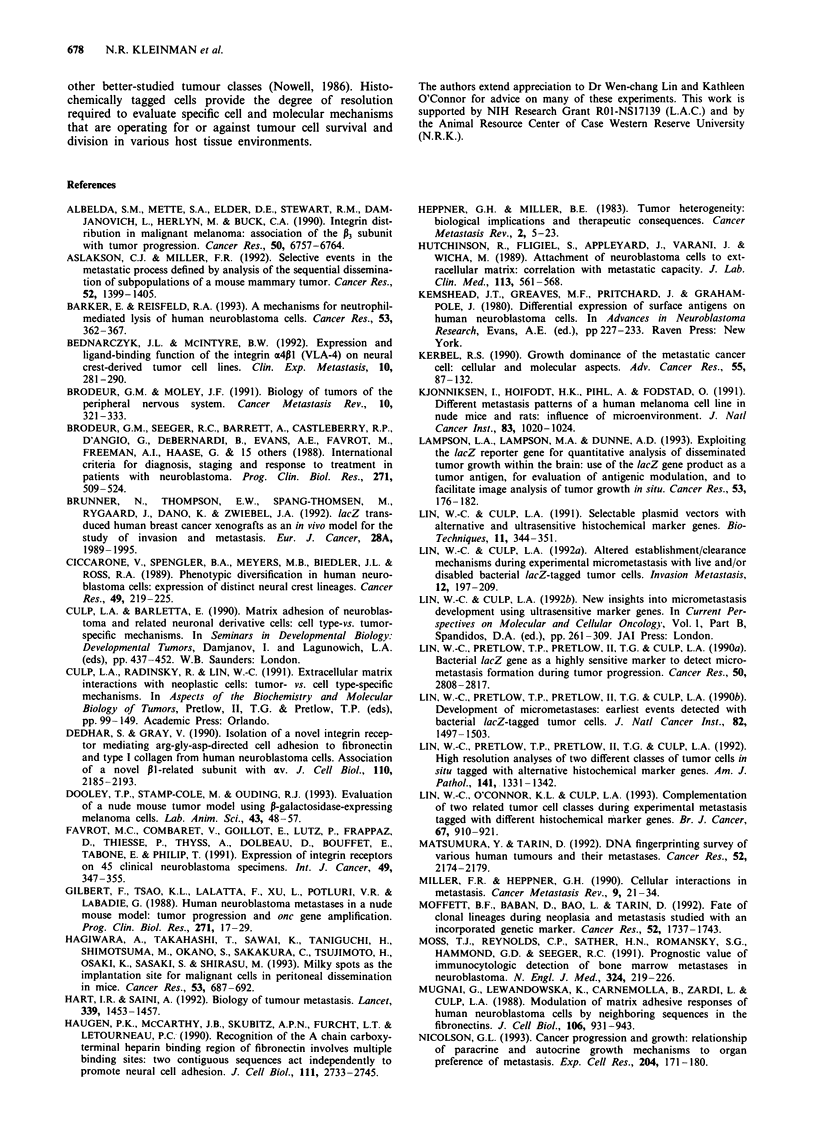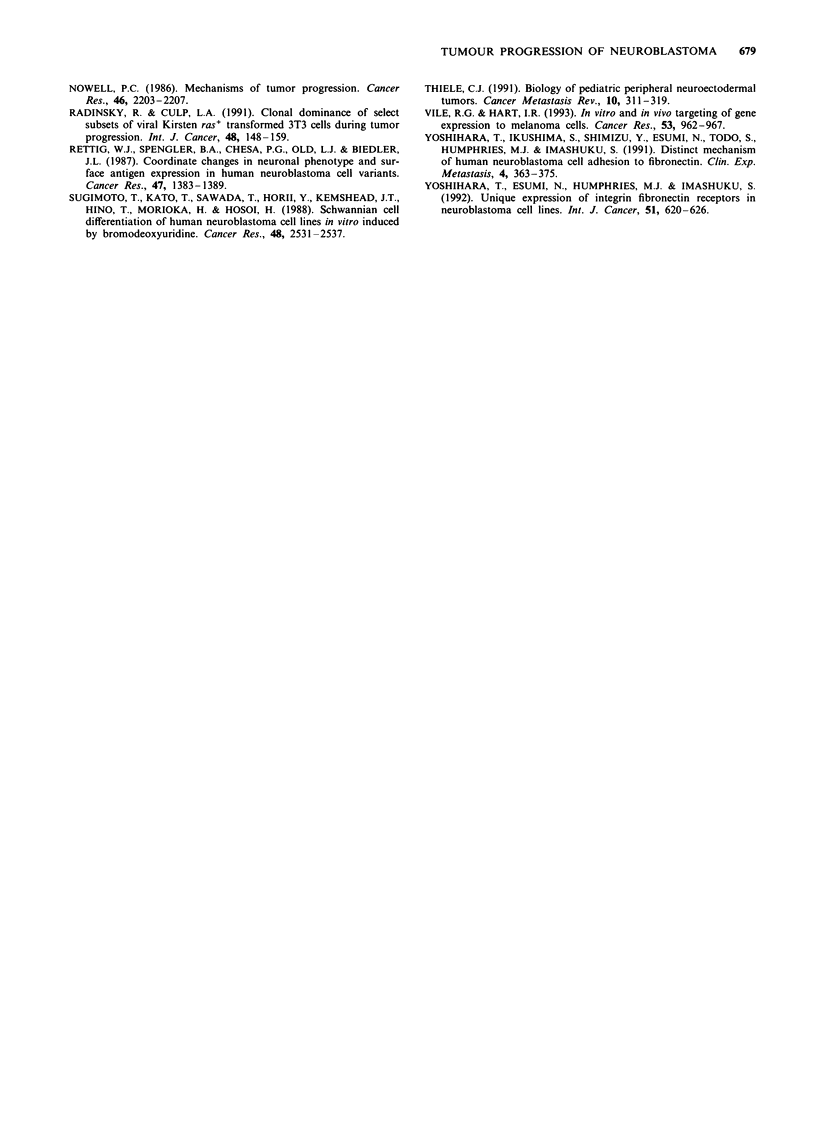# Tumour progression of human neuroblastoma cells tagged with a lacZ marker gene: earliest events at ectopic injection sites.

**DOI:** 10.1038/bjc.1994.129

**Published:** 1994-04

**Authors:** N. R. Kleinman, K. Lewandowska, L. A. Culp

**Affiliations:** Department of Molecular Biology and Microbiology, Case Western Reserve University, School of Medicine, Cleveland, Ohio 44106.

## Abstract

**Images:**


Br. J. Cancer (1994), 69, 670-679 © Macmillan Press Ltd., 1994

### Tumour progression of human neuroblastoma cells tagged with a *lacZ* marker gene: earliest events at ectopic injection sites

N.R. Kleinman[1,2], K. Lewandowska[1] & L.A. Culp[1]

[1]*Department of Molecular Biology and Microbiology and* [2]*Animal Resource Center, Case Western Reserve University, School of Medicine, Cleveland, Ohio 44106, USA.*

**Summary** Human Platt neuroblastoma cells were transfected with the marker gene, bacterial *lacZ*, to track cells at the earliest stages after ectopic injection at two different sites in athymic nude mice. Three clones (LZPt-1,-2 and -3) of differing morphologies were analysed. All clones yielded large primary tumours subcutaneously or intradermally with similar latency. While LZPt-2 and -3 clones generated well-staining primary tumours, LZPt-1 cells yielded many non-staining tumours, indicating greater instability of *lacZ* expression for this clone *in situ* (stability of *lacZ* expression in culture was similar for all three clones). After s.c. or intradermal injections, tumour cells were tracked for 1 h to >3 weeks (palpable) to evaluate the topology and population expansion characteristics at the earliest times. From 1 h to 2 days, tumour cells were concentrated in central masses with 'crinkly hair' distributions emanating from the periphery. Between 3 and 7 days, these 'crinkly hair' patterns were cleared from the tissue, leaving dense ovoid patterns of tumour cells. These concentrations of cells expanded collectively, not by division of one or a few cells, but by division of many cells. For clone LZPt-1, cells stained well with X-gal for 2-3 days; by 7 days, most cells were non-staining. Evidence suggests that *lacZ* expression is turned off in these tumour cells, rather than a *lacZ*$^-$ cell type clonally dominating the population. For all three clones, tumour cells remained rounded and did not spread in any tissue environment at all time points, indicating very different matrix adhesion mechanisms operating *in situ* compared with their distinctive spreading patterns in culture. Angioneogenesis near primary tumours became evident by 2-3 days, leading to extensive vascularisation by 1-2 weeks. Overall, these studies indicate common tumour progression characteristics for three different clones of human neuroblastoma, insight into *lacZ* instability mechanisms operating in one of these clones and the earliest events in primary tumour formation for this tumour at two different ectopic sites.

Progression of human neuroblastoma tumours from the initial stage of transformation of an embryonic precursor cell in the neural crest to advanced stages of metastasis in the nervous system, the adrenal gland and bone marrow has not been well studied in many regards (Brodeur *et al.*, 1988; Gilbert *et al.*, 1988; Brodeur & Moley, 1991; Moss *et al.*, 1991; Thiele, 1991). Little is known about clonal selections that occur at various stages of tumour progression, the routes of tumour cell spread during metastasis, critical genes required for progression, extracellular matrix mechanisms important in these events and host cell populations that promote or inhibit these processes. Analyses of extracellular matrix adhesion of neuroblastoma cells in culture revealed mechanisms distinct from those seen in many other cell types. Neuroblastoma cells recognise sequences on fibronectin different from most untransformed cells that have conventional integrin and heparan sulphate proteoglycan receptors (Mugnai *et al.*, 1988; Culp & Barletta, 1990; Culp *et al.*, 1991). These tumour cells can be missing many of the common integrin subunits (Favrot *et al.*, 1991; Yoshihara *et al.*, 1991, 1992) and in other cases have a unique form of the $\beta_1$ subunit (Dedhar & Gray, 1990). Some neuroblastoma cells express the integrin $\alpha_4\beta_1$ (Haugen *et al.*, 1990; Bednarczyk & McIntyre, 1992), used by some lymphoid cell classes for cell recognition functions but not by most adherent cells in the body; this integrin appears in an altered form in some cases (Bednarczyk & McIntyre, 1992). Some adhesion properties in culture correlate with metastatic spread *in situ* (Hutchinson *et al.*, 1989).

To relate the significance of findings in culture model systems to actual tumour progression and metastasis processes *in situ*, human neuroblastoma cells must be identifiable in host organs in a nude mouse experimental system. To facilitate detection of tumour cells at the single-cell level, our laboratory developed use of the bacterial *lacZ* gene, transfected into *ras*-transformed fibroblasts, as a genetic and histochemical marker to track fibrosarcoma cells staining intensively blue upon X-gal treatment (Lin *et al.*, 1990*a*, *b*). Alternative histochemical marker genes - *Drosophila* alcohol dehydrogenase or human placental alkaline phosphatase - were also developed to genetically tag two or more tumour cell classes to evaluate metastatic co-routing in virtually any host tissue (Lin & Culp, 1991). Co-injection of *ras*- or *sis*-transformed fibroblasts, tagged with different histochemical marker genes, demonstrated intercellular cooperation during the earliest events in experimental metastasis by two closely related tumour cell derivatives (Lin *et al.*, 1992, 1993), as well as insight into some aspects of tumour cell clearance in target organs (Lin *et al.*, 1990*b*; Lin & Culp, 1992*a*). The *lacZ* marker gene has now been used to tag breast carcinoma cells (Brunner *et al.*, 1992), melanoma cells (Dooley *et al.*, 1993) and glioblastoma cells (Lampson *et al.*, 1993), as well as to track therapeutic 'targeting' genes to tumour cell populations *in situ* (Vile & Hart, 1993).

In these studies, we analyse the earliest fates of human neuroblastoma cells injected into athymic nude mice at two different ectopic sites using the bacterial *lacZ* marker gene transfected into cells. Three stably expressing clones with morphologies characteristic of N-type, S-type and I-type neuroblastoma cells were studied (Rettig *et al.*, 1987; Ciccarone *et al.*, 1989). These analyses demonstrate several important findings with regard to the fates of injected cells and expansion of tumour cell populations with time at ectopic sites, including different matrix recognition *in situ* from those in culture and utility of the *lacZ* marker gene to evalute genetic instability in tumour cell populations during their expansion.

### Materials and methods

### *Generation of LZPt cell lines*

Cells, free of *Mycoplasma*, were grown in Dulbecco's modified Eagle medium supplemented with antibiotics and 10% neonatal calf serum. Human Platt neuroblastoma cells (Kemshead *et al.*, 1980) were transfected with the pRSV/*lacZ* plasmid, which also codes for the *neo*$^R$ gene using the calcium

Correspondence: L.A. Culp.
Received 6 August 1993; and in revised form 12 November 1993.

phosphate protocol (Lin & Culp, 1991) under conditions which maximise expression of the bacterial marker gene (Lin *et al.*, 1990*a,b*). Stably transfected/expressing cells were selected with G418 in the medium and G418-resistant colonies tested for *lacZ* expression, i.e. staining intensively blue with X-gal treatment. High-expressing colonies were cloned twice sequentially to generate three cloned populations (abbreviated to LZPt-1, -2, and -3 and selected for morphological criteria and for their uniform *lacZ* expression, as determined both by blue staining with X-gal substrate and by FACS using fluorescein digalactoside substrate uptake and conversion in living cells; Lin & Culp, 1992*b*). Non-transfected tumour cells failed to stain at all. LZPt clones harbour one copy of the *lacZ* gene and in culture remain uniformly stainable with X-gal for $>15$ passages; they gradually lose stainability with continuous culture over long periods of time by overgrowth of *lacZ*-non-expressing variants. For tumour analyses, all cells were used within 15 culture passages of isolation.

*Animal tumour studies*

Animal use was approved by the Animal Care and Use Committee of Case Western Reserve University and executed in the AAALAC-accredited Athymic Animal Facility. Athymic nude mice (NIH nu/nu; 4–6 weeks old) were injected at indicated sites with $1 \times 10^5$ cells in phosphate-buffered saline (PBS). Each clone of tumour cells was injected at two sites per mouse (bilaterally and symmetrically) s.c. or intradermally; these sites included the hypochondriac, lateral and inguinal regions of the abdomen. For s.c. or intradermal injections, mice were anaesthetised with ketamine hydrochloride ($200\ mg\ kg^{-1}$) and acetylpromazine ($2\ mg\ kg^{-1}$) intraperitoneally prior to cell injection. Tumour cells were injected s.c. in 0.1 ml of PBS; intradermal injections used 0.025 ml of PBS-suspended cells. To identify injection sites easily, skin was tattooed intradermally with two foci of India ink particles flanking the injection site; tattoos persisted for periods in excess of 6 weeks. Mice were killed at the indicated time points by overdose of pentobarbital administered i.p. When the mice reached a surgical plane of anaesthesia, the thorax was opened and 5 ml of fixation solution [2% (v/v) formaldehyde/0.2% (v/v) glutaraldehyde in PBS] was injected slowly into the left ventricle of the heart while the right ventricle was incised to permit drainage. Skin and body wall sites of the ventral abdomen encompassing the India ink tattoos were harvested as a unit and placed in additional fixation solution for 12–24 h.

*Preparation of tissues for staining*

After fixation, skin samples and internal organs were stained for *lacZ* activity immediately (Lin *et al.*, 1990*a*, *b*). Skin sites with adherent body wall tissue were divided along the ventral midline prior to staining. One-half of each tissue, containing one injection site for each clone, was split along the subcutaneous fascial plane separating the skin from the underlying body wall. The opposite half containing a second set of injection sites was cut, using the India ink spots as a guide, into approximately 1-cm-diameter pieces, each of which included an injection site, associated skin, subcutaneous space and adjacent body wall tissue. For transverse sections, each of these pieces was also cut with a scalpel blade at the midpoint between India ink tattoos to traverse the site of injection.

*Histochemical staining of tissues*

All cultured cells and tissues were histochemically stained for bacterial β-galactosidase activity after glutaraldehyde/formaldehyde fixation as previously described (Lin *et al.*, 1990*a*, 1992). Cultured cells and tissues were incubated at 37°C for 12–24 h in stain solution: $1\ mg\ ml^{-1}$ X-gal, 20 mM potassium ferricyanide, 20 mM potassium ferrocyanide, 2 mM magnesium chloride in PBS. Organs from animals not injected with LZPt cells failed to stain by this protocol. Photomicrographs of organs and tissue specimens were obtained with a Nikon SMZU dissecting microscope equipped with a Microflex UFX using Fujichrome 64T film.

*Materials*

Culture flasks and cluster dishes were purchased from Becton Dickinson Labware (Oxnard, CA, USA); neonatal calf serum from Biologos (Naperville, IL, USA); Dulbecco's modified Eagle medium and G418 from Gibco (Grand Island, NY, USA); X-gal (5-bromo-4-chloro-3-β-indoyl-D-galactopyranoside) and fluorescein digalactoside from Research Organics (Cleveland, OH, USA); Permount and acetone from Fisher Scientific (Fairlawn, NJ, USA); glutaraldehyde from Eastman Kodak (Rochester, NY, USA); potassium ferricyanide, potassium ferrocyanide, paraformaldehyde, and formaldehyde from Sigma (St, Louis, MO, USA); ketamine hydrochloride and acetylpromazine from Fort Dodge Laboratories (Fort Dodge, IA, USA) and Ayerst Laboratories (New York, NY, USA) respectively; and pentobarbital from Ganes Chemicals (Pennsville, NJ, USA).

### Results

*In vitro characteristics of LZPt clones*

Three LZPt clones gave different morphologies in culture that persisted over the lifespan of experimental use (approximately 15 passages; data not shown). They had very high percentages of *lacZ* marker gene expression ($>98\%$ of cells assayed) during these passages using either X-gal staining to identify blue-staining cells under the light microscope or the substrate fluorescein digalactoside, whose product is detectable by FACS above the background intrinsic fluorescence of Platt cells (Lin & Culp, 1992*b*). LZPt-1 cells display an astrocytic morphology (Sugimoto *et al.*, 1988) with many short extending processes, resembling S-type neuroblastoma cells (Rettig *et al.*, 1987; Ciccarone *et al.*, 1989); LZPt-2 were highly biopolar and neuritogenic, resembling N-type cells; and LZPt-3 cells were heterogeneous in their morphology, containing a sizeable fraction of neuritogenic cells but also well-spread and astrocytic cells and can be classed as I-type cells (Rettig *et al.*, 1987; Ciccarone *et al.*, 1989).

*Tumorigenicity at the subcutaneous site*

It was first determined whether these three clones were tumorigenic at all in athymic nude mice. Each clone was injected s.c. and the size of tumour measured with time. For all three clones, latency of tumour detection varied from 25 to 30 days; all injected animals yielded tumours; and all foci grew rapidly into large tumours ($>1$ cm diameter) at all s.c. injection sites.

Expression of the *lacZ* marker gene was evaluated in large tumours (Figure 1). LZPt-2 cells yielded tumours completely stainable for *lacZ*, except where necrosis of the tumour was occurring (Figure 1a). The same was true for LZPt-3 cells (Figure 1b). For both clones, virtually all tumours retained *lacZ* expression throughout the lifespan of primary tumour development at the s.c. site. Retention of red cells in blood vessels during fixation of tissues permitted orientation of dermal blood vessels to sites of tumour development (Figure 1d and below). In addition, when s.c. tumours were carefully teased away from their locations at the surface of the animal, X-gal staining of the body wall side of the dermis revealed small populations of tumour cells generating 'footprints' of tumour that were implanting at the edge of the dermis (Figure 1d), including both large pools of tumour cells and small collections that were too numerous to count. Therefore, expression of the marker gene can be used to track tumour cell populations as they invade neighbouring tissues.

For LZPt-1 cells, a different result was obtained in most (but not all) large primary tumours. Most tumours failed to

**Figure 1** *lacZ* marker gene activity in large primary tumours. Each of the LZPt clones was injected into nude mice s.c. and primary tumours allowed to grow to diameters greater than 5 mm (rulers show 1 mm segments in **a**–**c**). These tumours and adjoining host tissue were harvested and stained with X-gal to identify regions of tumour where *lacZ* gene activity could still be detected. **a**, LZPt-2 tumour showing uniform X-gal staining throughout the tumour, except in some focal regions in the centre where necrosis was developing (magnification 35 ×). **b**, LZPt-3 tumour showing uniform X-gal staining (magnification 35 ×). **c**, This is an example of an LZPt-1 tumour in which most of the tumour fails to stain with X-gal (broad arrow in **c**) while a small population of tumour cells concentrated in one region of the tumour do stain (open arrow in **c**) (magnification 110 ×). **d**, A large subcutaneous LZPt-3 tumour was separated from the overlying skin of the animal and the skin was stained with X-gal to test for the presence of tumour cells (magnification 120 ×). (In animals not injected with tumour cells, the dermis was completely non-stainable.) Two different patterns of 'footprints' of tumour are shown on the dermal side of the subcutaneous space. Large collections of tumour cells are observed (large arrowheads), while small focal collections are numerous as 'footprints' (small arrowheads). Blood vessels are also very apparent throughout the dermis.

stain with X-gal when they had grown large (Figure 1c), indicating either that the *lacZ* gene was being turned off in a large fraction of these cells or that a variant clone, not expressing *lacZ*, was dominating this tumour at a very early time point (see below). It was also noted in large non-staining LZPt-1 tumours that small populations of stainable tumour cells could still be observed (open arrow in Figure 1c). Therefore, there are marked differences in stability of *lacZ* expression and/or clonal selectivity during primary tumour formation when comparing clone 1 with clone 2 or 3 cells (see below). The same characteristics of primary tumour development were observed when these clones were injected intradermally.

### *Earliest stages of primary tumour development at two ectopic sites*

Expression of *lacZ* permits detection of single tumour cells at virtually any organ site (Lin & Culp, 1992*b*). We take advantage of this sensitivity for tracking the development of primary tumours at the subcutaneous and intradermal sites for all three clones to determine the nature and extent of cell population expansions that occur at these earliest times. It should also provide indication as to how LZPt-1 primary tumours become enriched with *lacZ* non-expressing cells.

Figure 2 illustrates the distribution of LZPt-3 tumour cells at these two injection sites with time. As early as 1 h post injection, tumour cells are concentrated into a large mass of cells with some projections of cells, referred to as 'crinkly hair' distributions, that extend into host tissue sites along pathways of least resistance (Figure 2a1 and a2). By 48 h (Figure 2b1 and b2), these 'crinkly-hair' patterns are disappearing as a result of either clearance of tumour cells from host tissues or tumour cell migration back toward the central tumour mass. The former possibility is indicated by two pieces of evidence – lysis of these peripheral cells is evident with release of blue product into intercellular spaces and the absence of any polarity of cell movement (see below on this point). At intradermal sites, the topographical relationships between tumour cell populations and endogenous blood vessels is also apparent at these very early time points (Figure 2a2 and b2).

By 1 week, 'crinkly-hair' projections were completely absent (Figure 2c1 and c2) and the large number of cells in the central mass appeared to be expanding, not by division of one or a few cells but by division of many cells. Therefore, clearing of tumour cells at injection sites may be limited, and highly diluted tumour cells at the periphery are cleared most effectively. By 3 weeks, just before tumours become palpable, LZPt-3 sites had developed into large ovoid structures resulting from division of a large number of cells in the central mass. In all cases, there was no evidence that only one or a few cells were dividing to give rise to the primary tumour; rather, a very large number of cells were dividing to give rise to the tumour mass. In a few cases with LZPt-3, intradermal tumours were developing regions where the *lacZ* gene was not being expressed (arrowhead in Figure 2d2), a situation not noted for this clone at the subcutaneous site.

Tumours at intradermal sites were consistently associated with the dermis and did not spread across the subcutaneous space. In contrast, s.c. injections led to cell association ('footprints') with the dermal side of this space (e.g. Figure 1d), and

**Figure 2** Time course of early primary tumour formation at two sites. LZPt-3 cells ($10^5$) were injected either into subcutaneous sites identified by two intradermal spots of India ink particles for ease of identification (series 1 micrographs) or intradermally between the India ink spots (series 2 micrographs). Mice were then sacrificed at the indicated time points and skin/subcutaneous samples harvested as described in Materials and methods. $\mathbf{a_1}$, One hour post subcutaneous injection, dermis and associated subcutis (magnification 32 ×). $\mathbf{a_2}$, One hour post intradermal injection, dermis and associated subcutis (magnification 32 ×). $\mathbf{b_1}$, Forty-eight hours post subcutaneous injection, dermis and associated subcutis (magnification 32 ×). $\mathbf{b_2}$, Forty-eight hours post intradermal injection, dermis and associated subcutis (magnification 32 ×). $\mathbf{c_1}$, One week post subcutaneous injection, external abdominal oblique muscle, fascia and associated subcutis (magnification 43 ×). $\mathbf{c_2}$, One week post intradermal injection, dermis and associated subcutis (magnification 43 ×) $\mathbf{d_1}$, Three weeks post subcutaneous injection, external abdominal oblique muscle and parietal peritoneum (magnification 43 ×). $\mathbf{d_2}$, Three weeks post intradermal injection, dermis and associated subcutis (magnification 43 ×). Small arrowheads in photomicrographs $\mathbf{a_1}$, $\mathbf{a_2}$, $\mathbf{b_1}$ and $\mathbf{b_2}$ indicate 'crinkly-hair' patterns of multiple projections of tumour cell populations evident during the first 48 h of residence in these tissue sites. Small solid arrows in $\mathbf{a_2}$, $\mathbf{b_2}$ and $\mathbf{c_2}$ indicate small dermal blood vessels. Large solid arrows in $\mathbf{b_2}$, $\mathbf{c_2}$ and $\mathbf{d_2}$ indicate larger dermal blood vessels. Open arrows in $\mathbf{a_1}$ and $\mathbf{a_2}$ identify intradermal India ink spots. Large arrowhead in $\mathbf{d_2}$ labels area of a 3 week intradermal tumour with decreased *lacZ* expression, a phenomenon that is rare in large subcutaneous tumours using LZPt-3 cells.

by 1 week tumour expansion was associated with the fascia of the abdominal wall musculature. At intradermal injection sites, it was also common to observe tumour cells collected around blood vessels (e.g. Figure 2b2). These same early events were observed for LZPt-2 cells at both s.c. and intradermal sites (not shown).

Another perspective on early events was provided by transverse sectioning of the skin at the midline between the India ink tattoos, as shown in Figure 3 for LZPt-3 cells [the same findings with LZPt-2 cells (not shown)]. Transverse sections demonstrate accurate localisation of the injected tumour cells to the s.c. site (series 1 of Figure 3) or intradermal site (series 2). Because of the sensitivity of *lacZ* stainability, this approach provides appreciation for progression of primary tumours from early stages with 'crinkly hair' distributions (e.g. Figure 3a1 and a2), through the stage of consolidation and elimination of peripheral cells by 1 week (Figure 3b1 and b2), to the generation of a single spheroid tumour mass by 3 weeks (Figure 3c1 and c2). It also confirms that many tumour cells are dividing to give rise to the central tumour mass. It is also evident that tumour cells injected at the s.c. site do not invade the dermis at early time points; also, these cells injected intradermally do not traverse the subcutaneous space.

LZPt-1 cells are unique in that they generate large tumours consisting almost entirely of *lacZ* non-expressing cells (Figure 1c). The basis for this was investigated by tracking the distribution of expressing and non-expressing cells (Figure 4). In Figure 4a, b, e and f, LZPt-1 cells at intradermal sites are stainable with X-gal for periods greater than 48 h and display the 'crinkly-hair' distributions observed for the other two clones. However, by 1 week post injection (Figure 4c), the pattern of staining has changed dramatically under conditions in which the cell population has expanded only to a limited extent. X-gal staining is very weak in most tumour cells, with a diffuse blue pattern in the tissue as if *lacZ* expression is being turned off synchronously in most of the population (large arrowheads in Figure 4c). Only a few

**Figure 3** Transverse sections of LZPt-3 subcutaneous and intradermal tumours. Cells were injected as described in the legend to Figure 2 and Materials and methods. At the indicated times following subcutaneous (series 1 micrographs) or intradermal (series 2 micrographs) inoculation, animals were sacrificed and the injection sites were harvested. Transverse sections through epidermis, dermis, subcutis, abdominal wall musculature and parietal peritoneum were performed at the midline between the two India ink spots. $\mathbf{a_1}$, One hour post subcutaneous injection (magnification 87 ×). $\mathbf{a_2}$, One hour post intradermal injection (magnification 130 ×). $\mathbf{b_1}$, One week post subcutaneous injection (magnification 174 ×). $\mathbf{b_2}$, One week post intradermal injection (magnification 43 ×). $\mathbf{c_1}$, Three weeks post subcutaneous injection (magnification 87 ×). $\mathbf{c_2}$, Three weeks post intradermal injection (magnification 43 ×). Double-arrowed lines demarcate the extent of the epidermis and dermis in $\mathbf{a_1}$ and $\mathbf{a_2}$; large open arrows identify the ventral abdominal musculature.

well-staining LZPt-1 tumour cells are observed at this site at 1 week even though the site is composed of many thousands of cells (small arrowhead in Figure 4c). These results indicate that the expression of this marker gene in this particular clone is turned off by day 7 in most, but not all, tumour cells. By 3 weeks (Figure 4d and h), non-expressing cells have expanded in number and are contributing to the vast majority of tumour mass just prior to palpability. An exception to this pattern of non-stainable LZPt-1 tumours is shown in Figure 4g, in which the 1-week-old tumour retains some stainability.

During these analyses, earliest evidence for tumour-induced angiogenesis occurred at the 48 h time point, particularly at well-vascularised intradermal sites. In Figure 5a, numerous small blood vessels are branching both towards and away from the LZPt-3 tumour cell mass, originating from a major blood vessel near the injection site. These small vessels occur at very low frequency at other sites in the skin

**Figure 4** Loss of $\beta$-galactosidase activity of LZPt-1 cells at early time points. Photomicrographs **a**–**d** show dermis and associated subcutis, while photomicrographs **e**–**h** are transverse sections through epidermis, dermis, subcutis, abdominal wall musculature and parietal peritoneum at the midline between India ink spots. **a**, One hour post intradermal injection (magnification 32 ×). **b**, Forty-eight hours post intradermal injection (magnification 43 ×). **c**, One week post intradermal injection (magnification 43 ×). **d**, Three weeks post intradermal injection (magnification 43 ×). **e**, One hour post intradermal injection (magnification 43 ×). **f**, Twenty-four hours post intradermal injection (magnification 43 ×). **g**, One week post intradermal injection (magnification 43 ×). **h**, Three weeks post intradermal injection (magnification 43 ×). Small arrowheads in **a** and **b** indicate the 'crinkly-hair' effect of projections of tumour cell populations from the central mass of cells evident during the first 48 h of residence. Open arrows in **a**, **b** and **e** identify intradermal India ink spots. Large black arrowheads in **c**, **d** and **h** delineate the margins of poorly staining to non-staining regions of tumour; small black arrowheads in the same three photomicrographs identify very small foci of *lacZ*-expressing cells.

where tumour cells are not resident, suggestive of tumour cell induction of these smallest vessels. Alternatively, these vessels may exist at all sites but cannot be readily observed by these microscopic procedures unless they expand into larger vessels in proximity to tumour cells. At 2 weeks post-injection, larger vessels appear to be feeding the LZPt-3 tumour and to penetrate the tumour mass (Figure 5b). By 3 weeks, many blood vessels course over the surface of a LZPt-1 tumour as well (Figure 5c). Again, this 3-week-old LZPt-1 tumour has lost most of its stainability with X-gal, with the exception of a small area labelled by the white open arrow (Figure 5c) in which *lac*Z-expressing cells persist. These development patterns for blood vessels at the peripheries of tumours were noted for all three clones.

**Figure 5** Promotion of angiogenesis at early tumour cell foci. LZPt cells were injected intradermally or subcutaneously as indicated and tissues harvested for staining with X-gal. **a**, LZPt-3 cells 48 h post intradermal injection (magnification 87 ×). **b**, LZPt-3 cells 2 weeks post intradermal injection (magnification 87 ×). **c**, LZPt-1 cells 3 weeks post subcutaneous injection (magnification 43 ×). Small black arrows in all photomicrographs indicate small blood vessels that appear to be induced at the periphery of the primary tumour at these early time points. Large black arrows identify large blood vessels from which small vessels originate. Large solid arrowhead in **c** labels area of *lacZ* non-expression in 3 week tumour of LZPt-1 cells while the white open arrowhead indicates a small region of persistent stainability.

High resolution analyses on the Nikon SMZU microscope permitted evaluation of the morphologies of individual tumour cells. At earliest times, cells of all three clones at both injection sites were very rounded with minimal spreading in tissues, even though they spread well in culture at these times. Figure 6a illustrates this rounded morphology for LZPt-1 cells at 24 h at the s.c. site and Figure 6b for LZPt-3 cells 48 h at the intradermal site as they surround a major blood vessel. By 3 weeks when most of the LZPt-1 tumour had become non-expressing for *lacZ*, a few *lacZ*-expressing cells were observed to extend neurite-like processes in the tumour mass (small arrow in Figure 6c). In contrast, most of the LZPt-3 cells in a 2-week-old tumour persisted in their round morphology (Figure 6d). These results indicate that neuroblastoma cell-matrix interactions are very different *in situ* than they are in culture.

### Discussion

This study demonstrates that human neuroblastoma cells can be stably transfected with the bacterial *lacZ* gene, providing a histochemical marker to track tumour cells in any organ at the single-cell level. *lacZ* expression was stable in all three clones in culture for > 15 passages. In contrast, early-passage LZPt-1 cells yielded non-staining primary tumours in many, but not all, cases, while LZPt-2 and -3 clones yielded uniformly staining tumours, similar to *lacZ*-transfected fibrosarcoma cells (Lin *et al.*, 1990*a*, 1993). Therefore, *lacZ* affords the opportunity to evaluate instability in marker gene expression for tumour cells both quantitatively and qualitatively and by comparing *in vitro*-grown populations with tumour-progressing populations *in situ*.

Neuroblastoma clones of three different morphologies (N-, S- and I-type cells; Rettig *et al.*, 1987; Sugimoto *et al.*, 1988; Ciccarone *et al.*, 1989) display tumorigenicity in nude mice at s.c. sites similar to parental cells. At both s.c. and intradermal sites ectopic for neuroblastoma, all three clones yielded similar latency times and similar kinetics of primary tumour development. During expansion of s.c. tumours, small populations of tumour cells, referred to as ‘footprints’, developed on the dermal side of the s.c. space, demonstrating the earliest stages of primary tumour invasion of neighbouring tissues (Hagiwara *et al.*, 1993). Overall, these studies reveal many similarities and only a few subtle differences in the tumour progression characteristics among N-type, S-type (or Schwannian-type cells; Sugimoto *et al.*, 1988) and I-type cells.

*lacZ* enabled visualisation of tumour cells at earliest times after s.c. or intradermal injections to evaluate how cells localise in tissues, if and where tumour cell clearance occurs and how populations yield a palpable tumour. All three clones at both injection sites yield ‘crinkly-hair’ patterns at their edges for 2–3 days, presumably by cells penetrating along paths of least resistance in the host tissue. Tumour cells are then consolidated into concentrated populations by 7 days. Several pieces of evidence indicate that these ‘crinkly-hair’ populations are cleared from the tissue and that tumour cells do not migrate back towards the central tumour mass. These cells fail to spread their cytoplasm in tissues or reveal any polar migration pattern. Tumour cells in the ‘crinkly-hair’ also yield some diffuse blue stain spreading through the tissue, characteristic of tumour cell lysis (this was not observed after the consolidation phase). These results indicate some degree of tumour cell density dependence for survival. In this regard, neuroblastoma cells have been shown to be susceptible to neutrophil-mediated lysis (Barker & Reisfeld, 1993).

This LZPt-1 system was particularly effective for visualising morphologies of individual tumour cells at all stages of primary tumour evolution. Most cells of all three clones maintain a rounded morphology devoid of neuritic processes or cytoplasmic spreading at all time points. The absence of

**Figure 6** Cellular morphology at high resolution during primary tumour development. Each of the three LZPt clones was injected intradermally or subcutaneously as indicated. At various times, injection sites and adjacent tissues were harvested, fixed and stained with X-gal. Tumour cells at the margins of tumour development were then photographed at very high magnification on the SMZU microscope to evaluate the morphologies of single cells. **a**, LZPt-1 cells 24 h post s.c. injection (magnification 326 ×). Well-rounded cells are evident throughout the tissue (e.g. at the arrow). **b**, LZPt-3 cells 48 h post intradermal injection (magnification 326 ×). Well-rounded tumour cells (e.g. at the arrow) appear to surround this major blood vessel. **c**, LZPt-1 cells 3 weeks post intradermal injection (magnification 326 ×). While most tumour cells are non-staining (large arrowhead), a few tumour cells do stain for *lacZ*, some of which extend neurite processes in the tumour (small arrow). **d**, LZPt-3 cells 2 weeks post s.c. injection (magnification 652 ×). Virtually all tumour cells remain rounded (e.g. small arrow) at all locations in this growing tumour.

spreading *in situ*, compared with their spreading into N-, S- and I-type cells in culture (Rettig *et al*., 1987; Ciccarone *et al*., 1989), provides further evidence for the distinctive tumour cell–matrix adhesion mechanisms operating in host tissues (Culp *et al*., 1991). In a few select cases, neurite extension of a few LZPt-1 cells could be observed in large primary tumours. This high-resolution system should permit more effective dissection of expression of specific genes associated with growth regulation of these tumour cells at their *in situ* locations. Some studies with melanoma cells demonstrate that integrin expression patterns change significantly at various stages of tumour progression (Albelda *et al*., 1990).

Finally, the primary tumour cell population expands significantly between 1 week and 3 weeks, when tumours become palpable. This raises the issue of clonal expansion of tumour cells to give rise to overt primary tumours. There was no evidence throughout this phase for only one or a few cells dividing to give rise to a clonally dominant population, as indicated in drug resistance marker selection studies in a few other tumour systems (Kerbel, 1990). Moreover, studies using gene markers have established in other tumour systems the clonal, phenotypic and genetic complexity of primary tumour populations during progression (Heppner & Miller, 1983; Miller & Heppner, 1990; Radinsky & Culp, 1991; Matsumura & Tarin, 1992; Moffett *et al*., 1992).

Evaluation of LZPt-1 tumour development provided insight into how these tumours become non-stainable by tracking the distribution of *lacZ*-expressing and non-expressing cells with time. Expression of the marker gene in this particular clone is lost by day 7 in most, but not all, tumour cells. These results indicate that non-stainability of LZPt-1 primary tumours cannot be explained by one cell losing expression of *lacZ* and subsequently overgrowing all its stainable neighbours; foci at 7 days are slightly larger than foci at 2 days and there would be insufficient time for one or a few non-staining cells to grow into such a large population. Rather, most cells become non-stainable apparently by some gene down-regulation mechanism that may be indicative of complex environmental control over gene expression in LZPt-1 cells, as suggested in other tumour systems (Kjonniksen *et al*., 1991; Aslakson & Miller, 1992; Hart & Saini, 1992; Nicolson, 1993).

Some insight into the earliest stages of tumour-induced angiogenesis were also revealed. Transcardial perfusion of fixation solution at euthanasia provided good resolution of blood vessels with their haemoglobin-containing cells and obviated the need for special endothelial cell staining. Angiogenesis was best visualised with intradermal tumours as the density of blood vessels is far greater in the dermis than in the hypodermis. Two days post injection was the earliest time that angioneogenesis could be observed; multiple small vessels, originating from larger dermal blood vessels in the vicinity of the injection site, were noted branching toward the tumour cell mass. By 1 week post injection, the vessels growing in the direction of the tumour had enlarged, while those growing in the opposite direction had disappeared.

Overall, these analyses illustrate the complexity of neuroblastoma tumour cell survival and population expansion after several different ectopic injections. They also reveal the specificity and distinctiveness of the tumour progression properties of this tumour class when compared with those of other better-studied tumour classes (Nowell, 1986). Histochemically tagged cells provide the degree of resolution required to evaluate specific cell and molecular mechanisms that are operating for or against tumour cell survival and division in various host tissue environments.

The authors extend appreciation to Dr Wen-chang Lin and Kathleen O'Connor for advice on many of these experiments. This work is supported by NIH Research Grant R01-NS17139 (L.A.C.) and by the Animal Resource Center of Case Western Reserve University (N.R.K.).

**References**

ALBELDA, S.M., METTE, S.A., ELDER, D.E., STEWART, R.M., DAMJANOVICH, L., HERLYN, M. & BUCK, C.A. (1990). Integrin distribution in malignant melanoma: association of the $\beta_3$ subunit with tumor progression. *Cancer Res.*, **50**, 6757–6764.

ASLAKSON, C.J. & MILLER, F.R. (1992). Selective events in the metastatic process defined by analysis of the sequential dissemination of subpopulations of a mouse mammary tumor. *Cancer Res.*, **52**, 1399–1405.

BARKER, E. & REISFELD, R.A. (1993). A mechanisms for neutrophil-mediated lysis of human neuroblastoma cells. *Cancer Res.*, **53**, 362–367.

BEDNARCZYK, J.L. & MCINTYRE, B.W. (1992). Expression and ligand-binding function of the integrin α4β1 (VLA-4) on neural crest-derived tumor cell lines. *Clin. Exp. Metastasis*, **10**, 281–290.

BRODEUR, G.M. & MOLEY, J.F. (1991). Biology of tumors of the peripheral nervous system. *Cancer Metastasis Rev.*, **10**, 321–333.

BRODEUR, G.M., SEEGER, R.C., BARRETT, A., CASTLEBERRY, R.P., D'ANGIO, G., DEBERNARDI, B., EVANS, A.E., FAVROT, M., FREEMAN, A.I., HAASE, G. & 15 others (1988). International criteria for diagnosis, staging and response to treatment in patients with neuroblastoma. *Prog. Clin. Biol. Res.*, **271**, 509–524.

BRUNNER, N., THOMPSON, E.W., SPANG-THOMSEN, M., RYGAARD, J., DANO, K. & ZWIEBEL, J.A. (1992). *lacZ* transduced human breast cancer xenografts as an *in vivo* model for the study of invasion and metastasis. *Eur. J. Cancer*, **28A**, 1989–1995.

CICCARONE, V., SPENGLER, B.A., MEYERS, M.B., BIEDLER, J.L. & ROSS, R.A. (1989). Phenotypic diversification in human neuroblastoma cells: expression of distinct neural crest lineages. *Cancer Res.*, **49**, 219–225.

CULP, L.A. & BARLETTA, E. (1990). Matrix adhesion of neuroblastoma and related neuronal derivative cells: cell type-*vs.* tumor-specific mechanisms. In *Seminars in Developmental Biology: Developmental Tumors*, Damjanov, I. and Lagunowich, L.A. (eds), pp. 437–452. W.B. Saunders: London.

CULP, L.A., RADINSKY, R. & LIN, W.-C. (1991). Extracellular matrix interactions with neoplastic cells: tumor- *vs.* cell type-specific mechanisms. In *Aspects of the Biochemistry and Molecular Biology of Tumors*, Pretlow, II, T.G. & Pretlow, T.P. (eds), pp. 99–149. Academic Press: Orlando.

DEDHAR, S. & GRAY, V. (1990). Isolation of a novel integrin receptor mediating arg-gly-asp-directed cell adhesion to fibronectin and type I collagen from human neuroblastoma cells. Association of a novel β1-related subunit with αv. *J. Cell Biol.*, **110**, 2185–2193.

DOOLEY, T.P., STAMP-COLE, M. & OUDING, R.J. (1993). Evaluation of a nude mouse tumor model using β-galactosidase-expressing melanoma cells. *Lab. Anim. Sci.*, **43**, 48–57.

FAVROT, M.C., COMBARET, V., GOILLOT, E., LUTZ, P., FRAPPAZ, D., THIESSE, P., THYSS, A., DOLBEAU, D., BOUFFET, E., TABONE, E. & PHILIP, T. (1991). Expression of integrin receptors on 45 clinical neuroblastoma specimens. *Int. J. Cancer*, **49**, 347–355.

GILBERT, F., TSAO, K.L., LALATTA, F., XU, L., POTLURI, V.R. & LABADIE, G. (1988). Human neuroblastoma metastases in a nude mouse model: tumor progression and *onc* gene amplification. *Prog. Clin. Biol. Res.*, **271**, 17–29.

HAGIWARA, A., TAKAHASHI, T., SAWAI, K., TANIGUCHI, H., SHIMOTSUMA, M., OKANO, S., SAKAKURA, C., TSUJIMOTO, H., OSAKI, K., SASAKI, S. & SHIRASU, M. (1993). Milky spots as the implantation site for malignant cells in peritoneal dissemination in mice. *Cancer Res.*, **53**, 687–692.

HART, I.R. & SAINI, A. (1992). Biology of tumour metastasis. *Lancet*, **339**, 1453–1457.

HAUGEN, P.K., MCCARTHY, J.B., SKUBITZ, A.P.N., FURCHT, L.T. & LETOURNEAU, P.C. (1990). Recognition of the A chain carboxy-terminal heparin binding region of fibronectin involves multiple binding sites: two contiguous sequences act independently to promote neural cell adhesion. *J. Cell Biol.*, **111**, 2733–2745.

HEPPNER, G.H. & MILLER, B.E. (1983). Tumor heterogeneity: biological implications and therapeutic consequences. *Cancer Metastasis Rev.*, **2**, 5–23.

HUTCHINSON, R., FLIGIEL, S., APPLEYARD, J., VARANI, J. & WICHA, M. (1989). Attachment of neuroblastoma cells to extracellular matrix: correlation with metastatic capacity. *J. Lab. Clin. Med.*, **113**, 561–568.

KEMSHEAD, J.T., GREAVES, M.F., PRITCHARD, J. & GRAHAM-POLE, J. (1980). Differential expression of surface antigens on human neuroblastoma cells. In *Advances in Neuroblastoma Research*, Evans, A.E. (ed.), pp 227–233. Raven Press: New York.

KERBEL, R.S. (1990). Growth dominance of the metastatic cancer cell: cellular and molecular aspects. *Adv. Cancer Res.*, **55**, 87–132.

KJONNIKSEN, I., HOIFODT, H.K., PIHL, A. & FODSTAD, O. (1991). Different metastasis patterns of a human melanoma cell line in nude mice and rats: influence of microenvironment. *J. Natl Cancer Inst.*, **83**, 1020–1024.

LAMPSON, L.A., LAMPSON, M.A. & DUNNE, A.D. (1993). Exploiting the *lacZ* reporter gene for quantitative analysis of disseminated tumor growth within the brain: use of the *lacZ* gene product as a tumor antigen, for evaluation of antigenic modulation, and to facilitate image analysis of tumor growth *in situ*. *Cancer Res.*, **53**, 176–182.

LIN, W.-C. & CULP, L.A. (1991). Selectable plasmid vectors with alternative and ultrasensitive histochemical marker genes. *BioTechniques*, **11**, 344–351.

LIN, W.-C. & CULP, L.A. (1992*a*). Altered establishment/clearance mechanisms during experimental micrometastasis with live and/or disabled bacterial *lacZ*-tagged tumor cells. *Invasion Metastasis*, **12**, 197–209.

LIN, W.-C. & CULP, L.A. (1992*b*). New insights into micrometastasis development using ultrasensitive marker genes. In *Current Perspectives on Molecular and Cellular Oncology*, Vol. 1, Part B, Spandidos, D.A. (ed.), pp. 261–309. JAI Press: London.

LIN, W.-C., PRETLOW, T.P., PRETLOW, II, T.G. & CULP, L.A. (1990*a*). Bacterial *lacZ* gene as a highly sensitive marker to detect micrometastasis formation during tumor progression. *Cancer Res.*, **50**, 2808–2817.

LIN, W.-C., PRETLOW, T.P., PRETLOW, II, T.G. & CULP, L.A. (1990*b*). Development of micrometastases: earliest events detected with bacterial *lacZ*-tagged tumor cells. *J. Natl Cancer Inst.*, **82**, 1497–1503.

LIN, W.-C., PRETLOW, T.P., PRETLOW, II, T.G. & CULP, L.A. (1992). High resolution analyses of two different classes of tumor cells *in situ* tagged with alternative histochemical marker genes. *Am. J. Pathol.*, **141**, 1331–1342.

LIN, W.-C., O'CONNOR, K.L. & CULP, L.A. (1993). Complementation of two related tumor cell classes during experimental metastasis tagged with different histochemical marker genes. *Br. J. Cancer*, **67**, 910–921.

MATSUMURA, Y. & TARIN, D. (1992). DNA fingerprinting survey of various human tumours and their metastases. *Cancer Res.*, **52**, 2174–2179.

MILLER, F.R. & HEPPNER, G.H. (1990). Cellular interactions in metastasis. *Cancer Metastasis Rev.*, **9**, 21–34.

MOFFETT, B.F., BABAN, D., BAO, L. & TARIN, D. (1992). Fate of clonal lineages during neoplasia and metastasis studied with an incorporated genetic marker. *Cancer Res.*, **52**, 1737–1743.

MOSS, T.J., REYNOLDS, C.P., SATHER, H.N., ROMANSKY, S.G., HAMMOND, G.D. & SEEGER, R.C. (1991). Prognostic value of immunocytologic detection of bone marrow metastases in neuroblastoma. *N. Engl. J. Med.*, **324**, 219–226.

MUGNAI, G., LEWANDOWSKA, K., CARNEMOLLA, B., ZARDI, L. & CULP, L.A. (1988). Modulation of matrix adhesive responses of human neuroblastoma cells by neighboring sequences in the fibronectins. *J. Cell Biol.*, **106**, 931–943.

NICOLSON, G.L. (1993). Cancer progression and growth: relationship of paracrine and autocrine growth mechanisms to organ preference of metastasis. *Exp. Cell Res.*, **204**, 171–180.

NOWELL, P.C. (1986). Mechanisms of tumor progression. *Cancer Res.*, **46**, 2203–2207.

RADINSKY, R. & CULP, L.A. (1991). Clonal dominance of select subsets of viral Kirsten *ras*$^{+}$ transformed 3T3 cells during tumor progression. *Int. J. Cancer*, **48**, 148–159.

RETTIG, W.J., SPENGLER, B.A., CHESA, P.G., OLD, L.J. & BIEDLER, J.L. (1987). Coordinate changes in neuronal phenotype and surface antigen expression in human neuroblastoma cell variants. *Cancer Res.*, **47**, 1383–1389.

SUGIMOTO, T., KATO, T., SAWADA, T., HORII, Y., KEMSHEAD, J.T., HINO, T., MORIOKA, H. & HOSOI, H. (1988). Schwannian cell differentiation of human neuroblastoma cell lines *in vitro* induced by bromodeoxyuridine. *Cancer Res.*, **48**, 2531–2537.

THIELE, C.J. (1991). Biology of pediatric peripheral neuroectodermal tumors. *Cancer Metastasis Rev.*, **10**, 311–319.

VILE, R.G. & HART, I.R. (1993). *In vitro* and *in vivo* targeting of gene expression to melanoma cells. *Cancer Res.*, **53**, 962–967.

YOSHIHARA, T., IKUSHIMA, S., SHIMIZU, Y., ESUMI, N., TODO, S., HUMPHRIES, M.J. & IMASHUKU, S. (1991). Distinct mechanism of human neuroblastoma cell adhesion to fibronectin. *Clin. Exp. Metastasis*, **4**, 363–375.

YOSHIHARA, T., ESUMI, N., HUMPHRIES, M.J. & IMASHUKU, S. (1992). Unique expression of integrin fibronectin receptors in neuroblastoma cell lines. *Int. J. Cancer*, **51**, 620–626.